# Supplementary material for: Discussing Human and Environmental Health Co-Benefits Related to Diet and Mobility Behaviours in the Primary Care Setting: A Qualitative Exploratory Study
Source: Int J Environ Res Public Health. 2025 Sep 30;22(10):1503. doi: 10.3390/ijerph22101503 (PMC12562415; doi:10.3390/ijerph22101503)
Supplement: Supplementary file 1 [file ijerph-22-01503-s001.zip › ijerph-3709603-supplementary.pdf]

Santé, viande et climat

Notre santé et celle des générations futures dépendent de notre environnement. Nous devons nous engager pour le protéger.

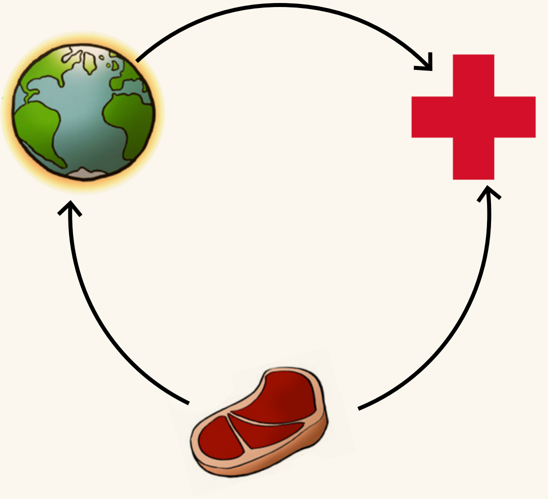

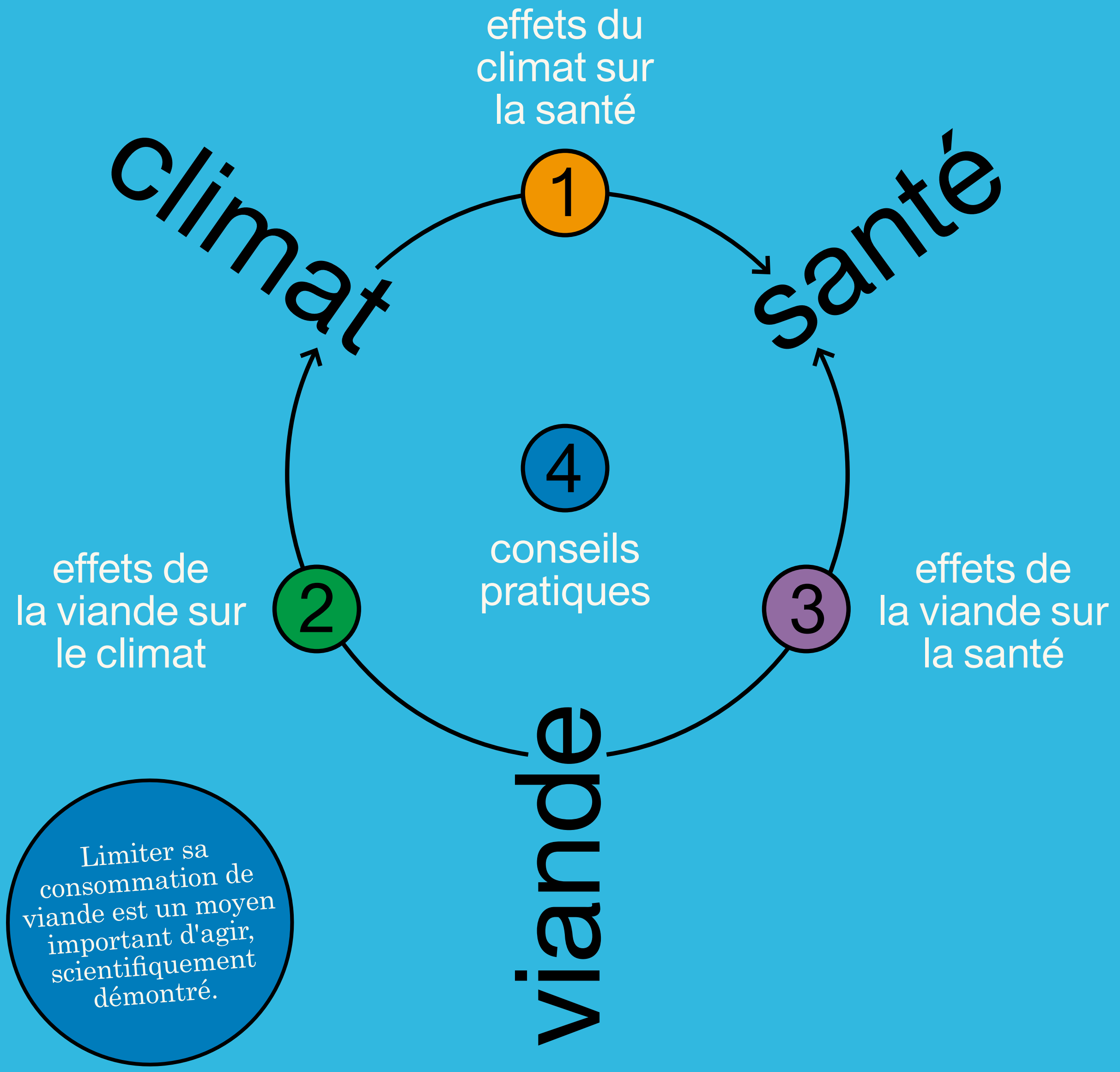

Engagés pour la Santé !
Groupe de propositions pour un système de santé durable

1 climat – santé

Les changements climatiques engendrent des coûts humains et économiques énormes.

Le changement climatique est la plus grande menace pour la santé globale au XXIe siècle.

OMS
Organisation Mondiale de la Santé

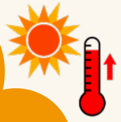

Canicules
2003 ~1000†
2015 ~800†

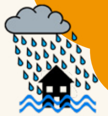

Inondations
2005 6†

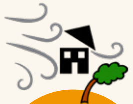

Des morts en Suisse

Tempêtes
1999 29†

Éboulements, laves torrentielles, coulées de boue
2000 13†
2017 8†
2019 2†

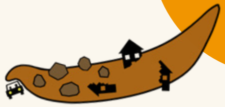

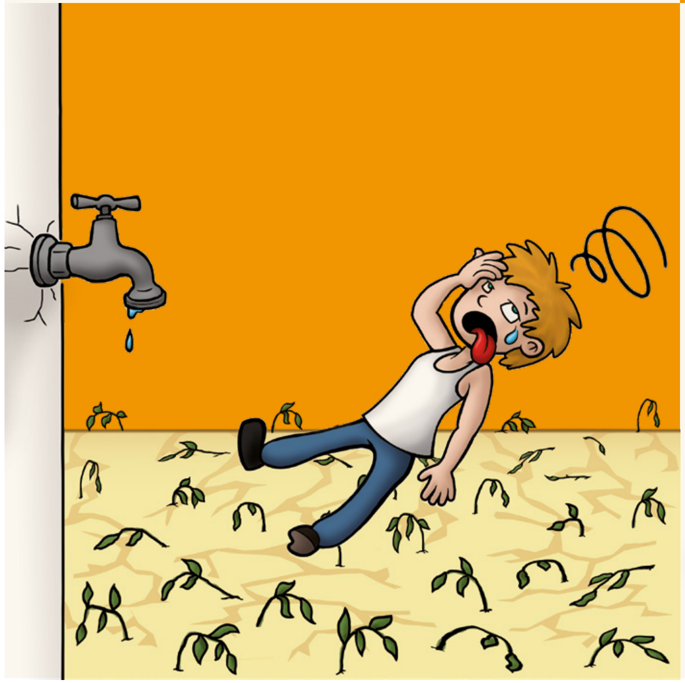

Un poids sur la santé physique et mentale

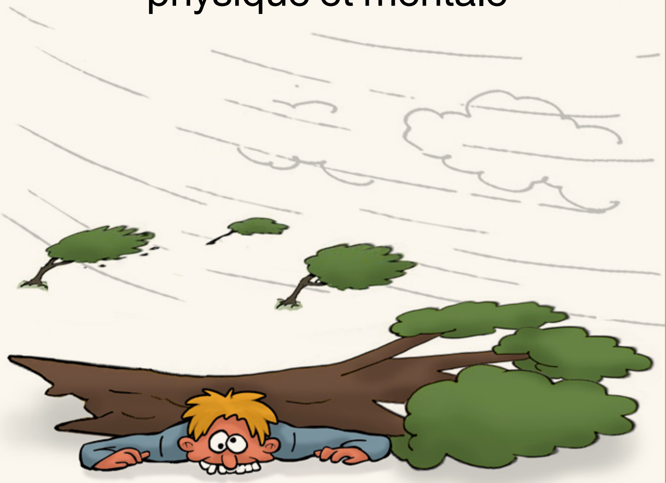

Les événements climatiques extrêmes sont déjà présents et vont se multiplier:

- sécheresses
- inondations
- vagues de chaleur
- feux de forêts
  etc.

La difficulté de pourvoir aux besoins de base augmente:

- alimentation
- air
- eau

Augmentation des problèmes de santé:

- cardiovasculaires
- respiratoires
- rénaux
- infectieux (maladies transmises par tiques et moustiques, diarrhées bactériennes…)
- psychiques
- nutritionnels

② viande – climat

« Consommer de la viande pèse lourd sur l'environnement. Face à la crise climatique il faut transformer notre système agro-alimentaire. » GIEC – Groupe d'experts intergouvernemental sur l'évolution du climat

L'élevage est responsable de 14 % des gaz à effet de serre mondiaux

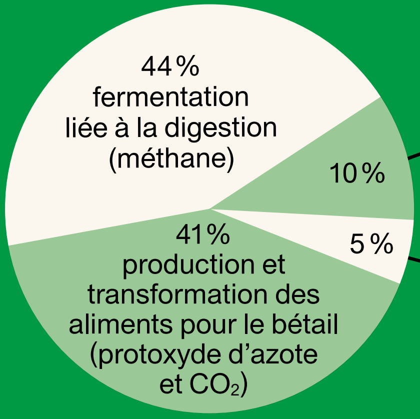

Le méthane a un potentiel de réchauffement climatique 25× et le protoxyde d'azote 300× plus élevé que le CO_2!

Équivalents CO_2 pour 1 kg de nourriture suisse

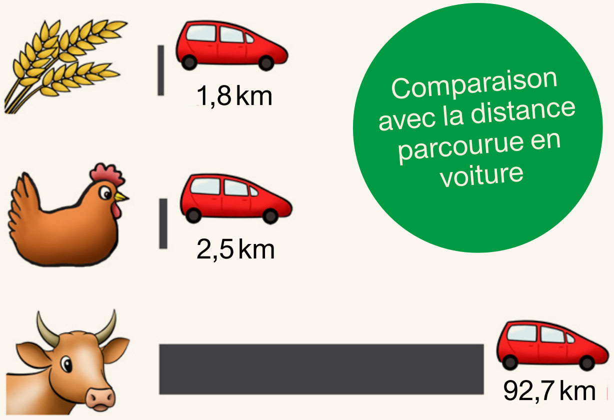

Comparaison avec la distance parcourue en voiture

Plus d'un quart de la charge environnementale suisse est due à l'alimentation

Près de la moitié de l'impact environnemental lié à l'alimentation est due aux produits d'origine animale!

La charge environnementale globale répertorie l'influence de la consommation sur l'ensemble des domaines environnementaux (climat, biodiversité, eau, air, etc.)

La viande

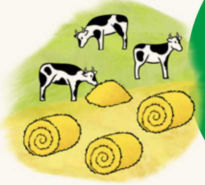

70 % de la surface agricole mondiale soit en pâturage soit pour produire la nourriture du bétail

91 % de la déforestation amazonienne pour les pâturages ou la production de nourriture

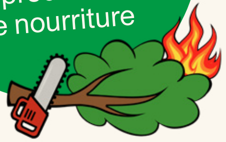

15'000 L d'eau pour produire 1 kg de bœuf

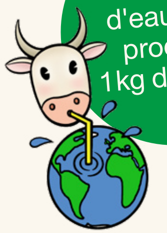

Diminuer sa consommation de viande est un geste efficace pour le climat

③ viande – santé

Remettons en question les croyances sur les bienfaits de la viande.

De beaux muscles rien qu'avec de l'herbe!

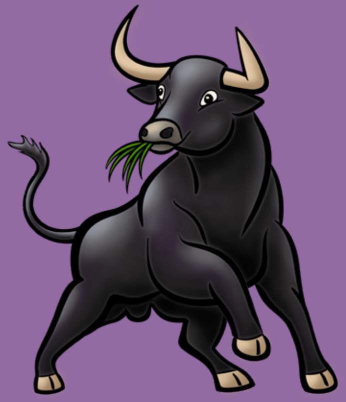

Chez l'humain,
les végétaux peuvent couvrir la majorité des besoins de l'organisme.

Une consommation de 3 portions au plus par semaine est bénéfique pour la santé:

- diminue le risque de cancer du colon
- diminue les risques de problèmes cardiovasculaires
- diminue les crises de goutte chez ceux qui y sont sujets

La production industrielle de viande a des conséquences sur:

- l'émergence de nouvelles zoonoses (la plupart des maladies infectieuses sont d'origine animale)
- l'augmentation de la résistance aux antibiotiques, qui est l'une des plus graves menaces actuelles sur la santé (OMS)
- la destruction de la biodiversité, des sols, de la forêt…

De plus, sur un plan éthique il n'est acceptable ni de maltraiter les animaux (95% des 25 millions de cochons français élevés par année ne voient pas la lumière du jour), ni de soumettre les ouvriers à des conditions de travail qui peuvent provoquer des états de stress post-traumatique dans les abattoirs.

Pour prendre soin de sa santé

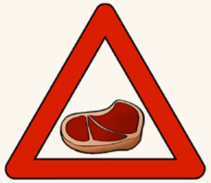

modérer
sa consommation
de viande

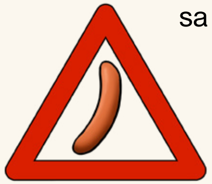

limiter sa
consommation
de charcuterie

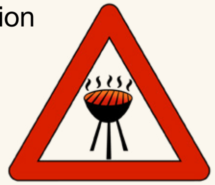

réserver
à l'occasionnel
la cuisson au grill

Se passer de viande? Attention aux nutriments

Nutriments prioritairement apportés par la viande et les produits d'origine animale:

- vitamine B12
- fer
- calcium
- zinc
- protéines
- oméga 3

Les **végétariens** ont globalement des avantages en terme de santé (souvent une bonne hygiène de vie, en plus de l'alimentation).

Les **vegans** nécessitent des connaissances poussées en nutrition + suppléments au moins en vitamine B12. Conseils médicaux recommandés.

Prudence particulière: enfants, femmes enceintes ou allaitantes, personnes âgées, lors de maladie.

4 conseils pratiques

L'alimentation est un levier important pour améliorer la santé et l'environnement : passons à l'action dès aujourd'hui

Conseils nutritionnels pour chacun

- favoriser légumineuses (lentilles, haricots, pois,…), oléagineux et céréales complètes en plus des légumes
- protéines végétales – associer légumineuses et céréales, dans le même repas ou la même journée
- fer – inclure au repas de la vitamine C (poivron, brocoli, choux, kiwi,…) Attention le thé, le café et les produits laitiers diminuent l'absorption du fer
- accorder une belle place à la viande blanche, consommer les morceaux moins nobles

Quelle quantité de viande pour ma santé ?

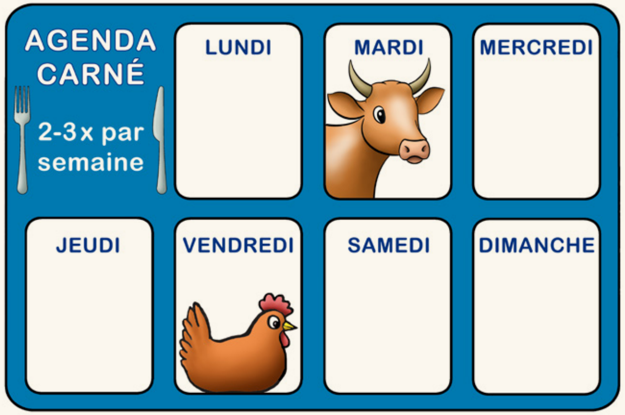

Consommer de la viande 2×/sem au lieu de 9 en moyenne actuellement en Suisse, diminuerait considérablement notre impact sur le climat

Implication au niveau politique

- adapter lois, taxes et subventions (utilisation des terres, produits alimentaires,…)
- informer et former la population et les professionnels (écoles, restaurations, hôpitaux…)
- favoriser les projets régionaux et nationaux (maraichage de proximité, pâturages,…)
- réduire le gaspillage (du producteur au consommateur)
- exemple: *Milan Urban Food Policy Pact* (209 villes européennes l'ont mis en œuvre Zürich, Lugano, Genève)

Principes de base consommer…

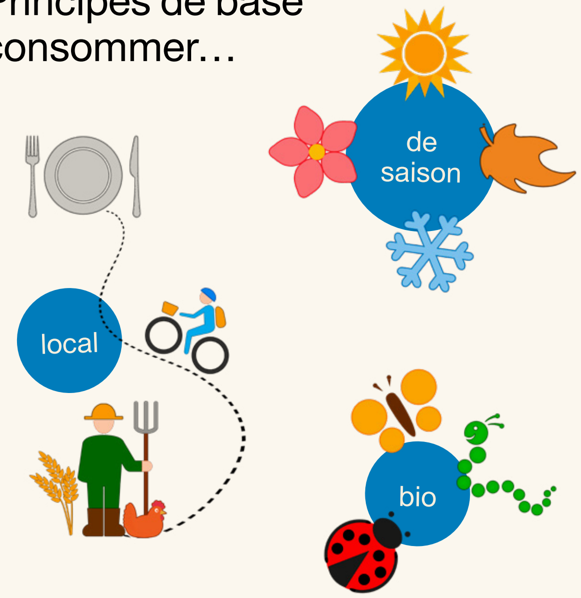

200 à 300g de viande par semaine c'est optimal pour la santé et adéquat pour le climat

Références et ressources

– BULLETIN NUTRITIONNEL SUISSE 2019 | Analyses de tendances relatives à l'utilisation des denrées alimentaires en Suisse, 2019

– ENVIRONMENTAL HEALTH: Impact of the warm summer 2015 on emergency hospital admissions in Switzerland, 2019 https://www.swisstph.ch/fr/actualites/heatwaves-in-crease-emergency-admissions-to-swiss-hospitals/

– FAO: FOOD AND AGRICULTURE ORGANISATION OF THE UN. Food Wastage Footprint – Impacts on Natural Resources – Summary Report, 2013 www.fao.org

– FAO ET OMS. Sustainable healthy diets: guiding principles, 2019
http://www.fao.org/3/ca6640en/ca6640en.pdf

– GIEC: GROUPE D'EXPERTS INTERGOUVERNEMENTAL SUR L'ÉVOLUTION DU CLIMAT. Rapports 1988 – révision 2019
www.ipcc.ch

– THE GUARDIAN. There's a Christmas crisis going on: no one wants to kill your dinner, novembre 2018
https://www.theguardian.com/commentisfree/2018/nov/19/christmas-crisis-kill-dinner-work-abattoir-industry-psychologi-cal-physical-damage

– IARC: INTERNATIONAL AGENCY FOR RESEARCH ON CANCER. Working Group on the Evaluation of Carcinogenic Risks to Humans. Red Meat and Processed Meat, 2018
https://monographs.iarc.fr/wp-content/uploads/2018/06/mono114.pdf

– LANCET EAT COMMISSION, 2019
1 www.thelancet.com/commissions/EAT
2 https://eatforum.org/content/uploads/2019/07/EAT-Lancet_ Commission_Summary_Report_French.pdf

– OFEV: OFFICE FÉDÉRAL DE L'ENVIRONNEMENT (Confédération suisse). Rapport sur l'environnement 2018 https://www.bafu.admin.ch/bafu/fr/home/documentation/rapports/rapport-environnement-2018.html

– OIE: ORGANISATION MONDIALE DE LA SANTÉ ANIMALE
https://www.oie.int/fr/

– OMS: ORGANISATION MONDIALE DE LA SANTÉ
https://www.who.int/fr/news-room/fact-sheets/detail/climate-change-and-health

– MILAN URBAN FOOD POLICY PACT
https://www.milanurbanfoodpolicypact.org/the-milan-pact/

– PHYSICIANS ASSOCIATION FOR NUTRITION PAN 2018
https://pan-int.org/pan-academy/

– PNR 69 SYNTHÈSE DU PROGRAMME NATIONAL DE RECHERCHE. Alimentation saine issue d'une production alimentaire durable
http://www.snf.ch/SiteCollectionDocuments/nfp/nfp69/PNR69_Synthese_brochure_FR_web.pdf

– Revue Médicale Suisse: https://www.revmed.ch
1 Changement climatique et enjeux cliniques, 2021
2 Cobénéfices santé et environnement: concepts et recommandations pour la pratique clinique, 2020
3 Co-bénéfices: Pourquoi introduire l'écologie dans la pratique clinique, 2020
4 Cobénéfices de la promotion de la santé sur le réchauffement climatique: L'exemple de l'alimentation et de la mobilité, 2020
5 Alimentations végétariennes et véganes: quelles conséquences
sur la santé, 2019

– SCIENCES 2018. Reducing food and environmental impacts through production and consumption:
https://science.sciencemag.org/content/360/6392/987

– SOCIÉTÉ SUISSE DE NUTRITION: www.sge-ssn.ch

VIDÉOS

– 4 minutes pour comprendre le vrai poids de la viande sur l'environnement. Le Monde.fr.
https://www.dailymotion.com/video/x2k2clo

– Les Damnés, des ouvriers en abattoir, documentaire d'Anne-Sophie Reinhardt, 2020
https://lesbatelieresproductions. com/films/l-arbre-qui- cachait-la-foret

– RTS. « Viande et climat sont conciliables, voici la recette des scientifiques », août 2019
https://www.rts.ch/info/economie/10653653-viande-et-climat-sont-conciliables-voici-la-recette-des-scient ifiques.html

Auteur.e.s

Engagés pour la santé
www.engagespourlasante.co
m

L'assiette selon EAT-Lancet

pour une alimentation saine et une production alimentaire durable

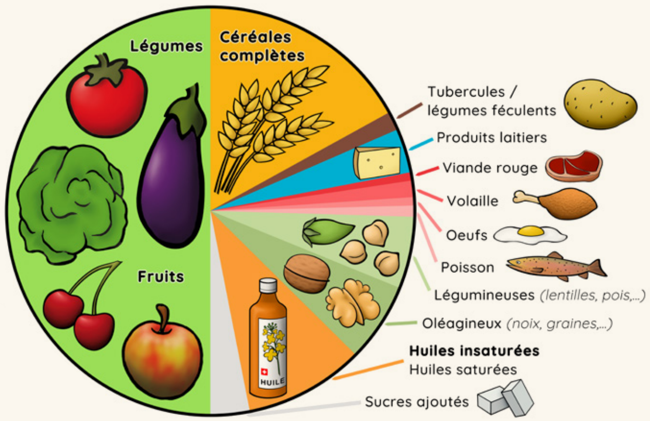

En volume
1re moitié : fruits et légumes
2e moitié : céréales complètes, protéines végétales, huiles végétales, +/- protéines animales en quantités modérées

Engagés pour la Santé
Groupe de propositions pour un système de santé durable

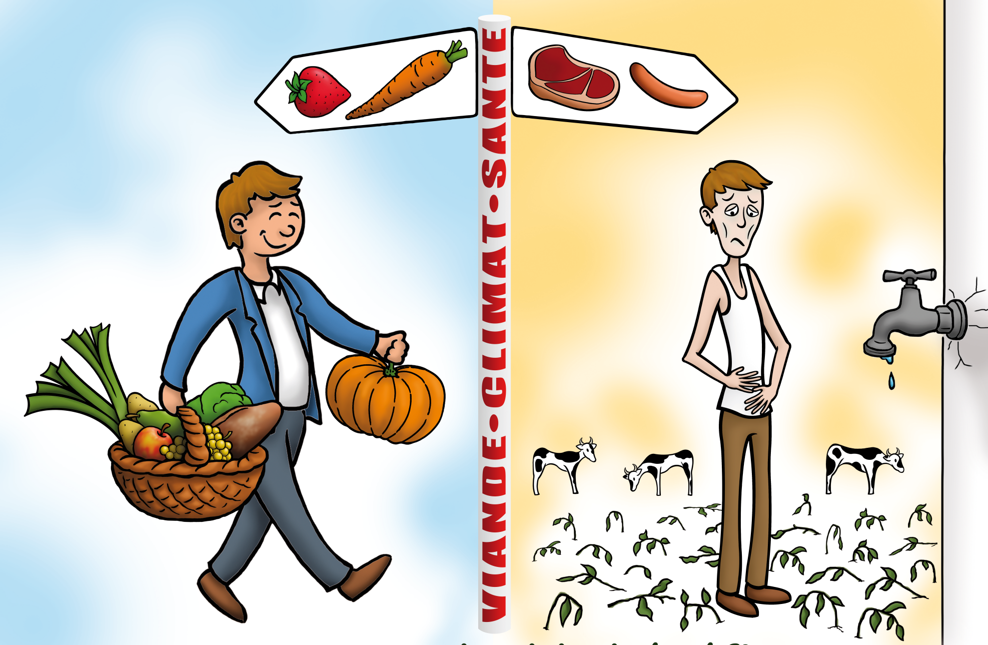

Un geste au double bénéfice : Modérer votre consommation de viande préserve votre santé et le climat !

3 portions de viande au plus par semaine suffisent à notre organisme. Trop de viande augmente les risques de cancer du colon et de problèmes cardiaques.

Notre santé dépend de notre environnement. Manger moins de viande, dont la production émet beaucoup de gaz à effet de serre, préserve climat et santé.

L'assiette selon EAT-Lancet

Pour une alimentation saine et une production alimentaire durable

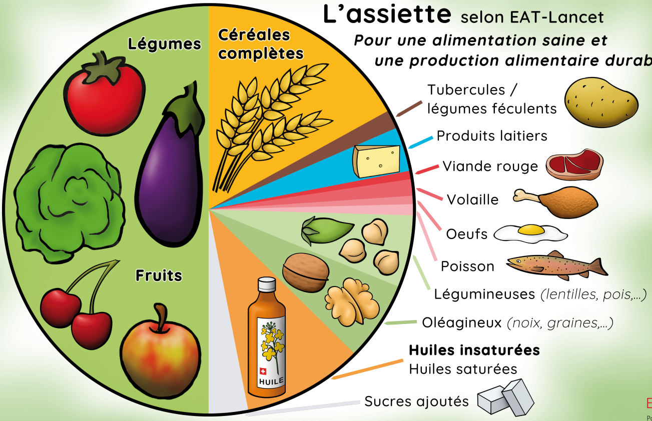

Conception V. Hof

Unil | Université de Lausanne – Centre de Compétences en Durabilité
Fondation pour l'Université de Lausanne
VOLTEFACE
unisanté – Centre universitaire de médecine générale et santé publique · Lausanne
Engagés pour la Santé ! – Pour un système de santé durable et équitable

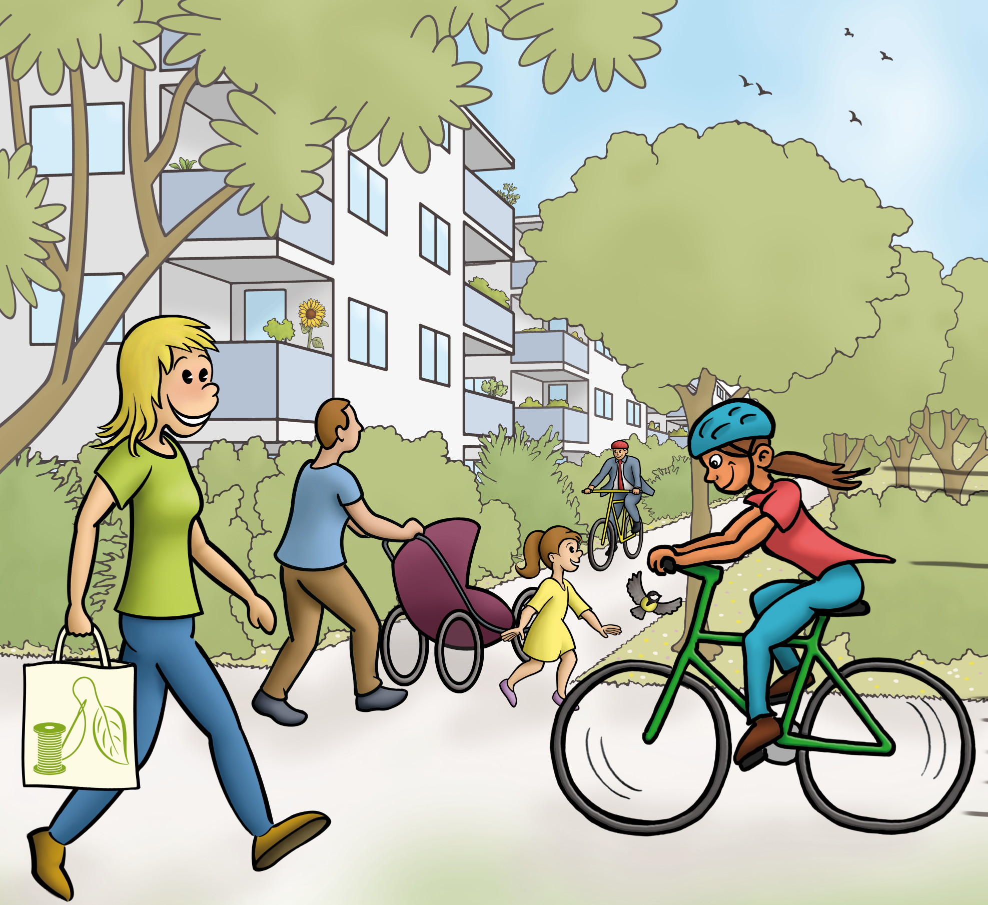

Un geste au double bénéfice :
En se déplaçant à pied ou à vélo
On prend soin de sa santé et du climat !

Une mobilité active régulière réduit efficacement les risques de maladies (problèmes cardiaques, diabète, cancer,...) et favorise la santé mentale.

Notre santé dépend de celle de notre environnement. Une réduction des déplacements motorisés préserve la qualité de l'air, le climat et ainsi la santé.

UNIL | Université de Lausanne
Centre de Compétences en Durabilité

FONDATION POUR L'UNIVERSITÉ DE LAUSANNE

VOLTEFACE

unisanté
Centre universitaire de médecine générale et santé publique • Lausanne

Engagés pour la Santé
Pour un système de santé durable et équitable

Conception V. Hof
